# SNORD126 Promotes Hepatitis C Virus Infection by Upregulating Claudin-1 via Activation of PI3K-AKT Signaling Pathway

**DOI:** 10.3389/fmicb.2020.565590

**Published:** 2020-09-15

**Authors:** Xijing Qian, Chen Xu, Bingan Wu, Hailin Tang, Ping Zhao, Zhongtian Qi

**Affiliations:** ^1^Department of Microbiology, Second Military Medical University, Shanghai, China; ^2^Spine Center, Department of Orthopedics, Changzheng Hospital Affiliated to Second Military Medical University, Shanghai, China

**Keywords:** small nucleolar RNA, non-coding RNA, SNORD126, hepatitis C virus, claudin-1, viral entry, ribonucleoprotein

## Abstract

Hepatitis C virus (HCV) infection involves a variety of viral and host factors, some of which promote the infection process. A small nucleolar RNA, C/D box 126 (SNORD126), was previously shown to be associated with hepatocellular carcinoma (HCC). However, the role of SNORD126 in HCV infection, which is one of the primary reasons for HCC development, has not been elucidated. In the present study, using small nucleolar RNA profiling, we observed that SNORD126 was significantly downregulated during HCV infection in both Huh7 and Huh7.5.1 cells. In addition, overexpression of SNORD126 enhanced HCV entry into host cells, whereas SNORD126 knockdown showed the opposite effect, suggesting that SNORD126 promotes HCV infection, especially through viral entry. Further functional analysis revealed that SNORD126 could enhance the expression level of claudin-1 (CLDN1), a key HCV entry factor, by increasing the levels of phosphorylated AKT. Additionally, the function of SNORD126 in HCV infection was associated with ribonucleoprotein (RNP) complexes. In summary, our findings demonstrate that oncogenic SNORD126 levels are decreased during HCV infection probably due to the host defense reaction, and SNORD126 may be important to promote viral entry by increasing CLDN1 expression through activation of the PI3K-AKT pathway, the mechanism of which is partly associated with SNORD126-mediated snoRNA RNP (snoRNP) function. Our work here provides initial evidence that endogenous snoRNA takes part in HCV infection and shows potential as a diagnostic or antiviral agent.

## Introduction

Hepatitis C virus (HCV) is responsible for almost 185 million hepatitis infections worldwide, and 50–80% of these cases can progress to chronic hepatitis and eventually develop into liver fibrosis, cirrhosis, and hepatocellular carcinoma (HCC) (Stanaway et al., 2016; Manns et al., 2017). As an important global public health problem, HCV results in billions of dollars in medical expenses and social resources every year (World Health Organization [WHO], 2017; Spearman et al., 2019; World Health Organization [WHO], 2020). Despite continued research, no optimal vaccine has been developed for HCV (Roingeard and Beaumont, 2020). Since 2011, the emergence of a number of direct-acting antivirals (DAAs), such as the NS3/4A protease inhibitor simeprevir, the NS5A inhibitor daclatasvir, and the NS5B polymerase inhibitor sofobuvir, have largely improved the efficacy of treatment for infected patients (Spengler, 2018; Ferreira et al., 2020). However, only a small number of HCV patients are diagnosed and treated with optimal DAA therapy. The naturally high mismatch rate during the HCV replication process and the low fidelity of its RNA polymerase allow this virus to continually develop drug resistance (Pawlotsky, 2016; Li and Chung, 2019). Moreover, whether DAAs reduce the incidence of HCV-related HCC remains controversial and needs further investigation, and antiviral treatments fail to prevent reinfection of patients with high risk factors (Simmons et al., 2016; Kanwal et al., 2017; Waziry et al., 2017). Thus, increased attention should be paid to elucidating the underlying host–virus interaction during HCV infection to solve this global problem.

HCV infection requires both virus and host factors, including endogenous genes, a large proportion of which are non-coding RNAs (ncRNAs). The identification and mechanistic elucidation of these stably and conservatively expressed RNAs will shed light on HCV gene diagnosis and therapy. Nevertheless, most studied ncRNAs in HCV are microRNAs, with numerous other types of ncRNAs being neglected, such as long non-coding RNAs (lncRNAs) or small nucleolar RNAs (snoRNAs) (Peng et al., 2009; Xiong et al., 2015). The results of our previous study demonstrated that an lncRNA called growth arrest–specific 5 (GAS5) can inhibit HCV replication by binding to viral NS3 protein, further confirming that different types of ncRNAs may play roles in HCV infection (Qian X. et al., 2016).

SnoRNAs comprise two families, including C/D box and H/ACA box RNAs, both of which are located in the nucleolus (Matera et al., 2007). SnoRNAs are 70–140 nucleotides in length and could form ribonucleoprotein (RNP) complexes with relevant proteins, which are the typical functioning mode of ncRNAs (Boivin et al., 2018). Most snoRNAs play roles in ribosomal RNA modification and processing, the modulation of RNA splicing and translation, and oxidative stress responses (Matera et al., 2007; Shuwen et al., 2020). Recently, an increasing number of studies have been carried out to elucidate the molecular mechanism of functional snoRNPs in human diseases, such as cancers or genetic disorders, as well as in many physiological processes (Stepanov et al., 2015; Cavaille, 2017; Li et al., 2017). One of our studies identified an H/ACA box snoRNA 7A (SNORA7A) as a crucial regulator during human umbilical cord mesenchymal stem cell proliferation and self-renewal, improving the clinical application of stem cell therapy (Zhang et al., 2017). However, few studies have investigated the specific modes of action of snoRNAs during viral infection despite their altered expression being frequently observed (Hutzinger et al., 2009; Saxena et al., 2013). SnoRNA, C/D box 126 (SNORD126) was first discovered through an advanced computational analysis of snoRNA genes in the human genome (Yang et al., 2006). Although the target and function of this snoRNA are unclear, it was previously studied as an oncogenic ncRNA in HCC and colorectal cancer (CRC) (Fang et al., 2017).

In our present study, we showed that SNORD126 was gradually suppressed along with HCV infection in both Huh7 and Huh7.5.1 cells by high-throughput sequencing analysis. Our results suggest that SNORD126 promotes HCV infection by upregulating the crucial viral entry factor claudin-1 (CLDN1) through activation of the PI3K-AKT pathway, probably by functioning as an snoRNP.

## Materials and Methods

### Cells Culture and Reagents

Human hepatoma Huh7 and Huh7.5.1 cells and human embryonic kidney HEK 293T cells were cultured in Dulbecco modified eagle medium (Life Technologies, United States) supplemented with 10% fetal bovine serum, 1 × non-essential amino acids, 100 IU/mL streptomycin and penicillin, and 2 mM L-glutamine (Gibco, Invitrogen). Cells were incubated under an atmosphere with 5% CO_2_ at 37°C for subsequent use in experimental procedures. Perifosine and SC79 were purchased from Selleck Chemicals (Houston, TX) and dissolved in dimethyl sulfoxide to generate 10 mM storage solutions for further dilution. Anti-HCV core, anti-SRB1, anti-CLDN1, anti-GAPDH antibodies, anti–pan-AKT, anti-pAKT(T308), and anti-pAKT(S473) primary antibodies were obtained from Abcam, whereas anti-CD81 and anti-OCLN antibodies and Alexa 488– and horseradish peroxidase–conjugated anti–rabbit and anti–mouse immunoglobulin G secondary antibodies were obtained from Invitrogen, with all antibodies used at appropriate dilution ratio according to the manufacturer’s instructions.

### High-Throughput Small RNA Sequencing and Bioinformatics Analysis

Huh7 and Huh7.5.1 cells were infected with JFH-1 cell culture–derived HCV (HCVcc), and total RNA was extracted at specific time points (0, 6, 24, and 48 h) postinfection using TRIzol reagent (Invitrogen, Carlsbad, United States) according to the manufacturer’s instructions for transcriptome sequencing. For small RNA sequencing, 10 μg of total RNA for each sample was used for small RNA cDNA library preparation as previously described with some modifications (Xu et al., 2016). Strand-specific RNA libraries were prepared using a TruSeq Small RNA Sample Prep kit (Illumina, San Diego, United States). Briefly, small RNA fragments ranging from 15 to 100 nt were isolated, purified, and subsequently ligated to 3′ and 5′ adaptors, reverse transcribed to cDNA, and then polymerase chain reaction (PCR) amplified. The entire library was tested by gel electrophoresis, and bands corresponding to microRNA insertion were cut and eluted. After ethanol precipitation and washing, the purified small RNA libraries were quantified and then sequenced using an Illumina HiSeq^TM^ 2000 analyzer (Illumina, San Diego, United States) according to the manufacturer’s instructions. All profiling assays were performed with the aid of Shanghai NovelBio Bio-Pharm Technology Co., Ltd.

For analysis of small RNA sequencing data, we first generated the clean reads and aligned then with the human genome (hg19) using Tophat2/Bowtie2, which was followed by calculating the distribution of reads in different regions of the genome. The clean reads were compared with the Rfam database^1^ to identify the known RNA sequences and were then compared with the human snoRNAs database in NCBI RefSeq to identify snoRNAs and count their reads. The raw counts of snoRNA reads were further normalized by TPM (transcripts per million) values [(snoRNA total reads/total clean reads) × 10^6^]. The differentially expressed snoRNAs between samples were identified with the program EdgeR using parameters of *P* ≤ 0.01 and fold change ≥ 2 or ≤ 0.5. The processed data are shown in Supplementary Data 1. Human chronic HCV-infected liver sample data of snoRNAs were derived from GEO dataset GSE87843, the raw data of which can be found in the GEO website^2^. The processed data for snoRNAs are shown in Supplementary Data 2.

### Plasmids, Antisense Oligonucleotides, and Small Interfering RNA Construction and Transfection

The full-length transcript cDNA of SNORD126 and its mutated transcript were synthesized and subcloned by Obio Technology Ltd. (Shanghai, China). The antisense oligonucleotides (ASOs) and small interfering RNAs (siRNAs) were synthesized for SNORD126 and CLDN1 knockdown (GenePharma Ltd., Shanghai, China). The sequences for all constructs are shown in Table 1.

**TABLE 1:** Oligonucleotide sequences used in this study.

SiRNA	Primer sequence, 5′–3′
	**Forward**

ASO-1	AACAUGCGGACUUAACAUGCAUUU
ASO-2	CUCAGAGCAUGUGUUUAAUCAGG
SICLDN1-1	GGGUGCUCCUUAAAUAUAU dTdT
SICLDN1-2	GGUGCUCCUUAAAUAUAUA dTdT
SINC	CCGCAGUCCUACUAGUUCA dTdT

Transfection was conducted with FuGene HD transfection reagent (Promega, Carlsbad, United States) according to the manufacturer’s protocols. Overexpression plasmid, ASOs, or siRNAs were incubated with transfection reagent in serum-free culture medium. Cells were then incubated with the transfection complexes for 24 h before changing the medium.

### Production of Cell Culture–Derived HCV and Infection Assay

HCVcc was generated as described previously (Wakita et al., 2005; Zhong et al., 2005). The Japanese fulminant hepatitis type 1 (JFH-1) plasmid, which was a gift from T. Wakita (National Institute of Infectious Diseases, Tokyo, Japan), was linearized and transcribed *in vitro* to produce HCV RNA using a MEGAscript kit (Promega, Madison, WI, United States). Then, 10^6^ Huh7 cells were transfected with 10 ng of viral RNA by electroporation. The cells were moved to culture plates for further viral amplification, and the medium was changed for fresh medium 4 h later. The supernatant was collected 96 h after transfection and filtered through a 0.45 μm membrane. HCVcc was then concentrated and purified by ultracentrifugation, and viral titers were determined for later experiments.

The infection assay was performed using Huh7 or Huh7.5.1 cells that were incubated with JFH-1 HCVcc at 37°C for 6 h before changing the medium. Cells were fixed 48 h postinoculation, and the infection was assessed by immunofluorescent (IF) assay with an anti-HCV core antibody.

### Production of HCV Pseudoparticles and Entry Assay

HCV pseudoparticle (HCVpp) was generated in accordance with a previous protocol (Qian X.J. et al., 2016). HEK 293T cells were transfected with packaging plasmids encoding viral envelope protein (1a strain of H77, a gift from F.L. Cosset, INSERM U758, Lyon, France), Gag/Pol, Rev, and pLenti6 transferring vector using Lipofectamine^TM^ 2000 (Invitrogen). The culture medium was exchanged for fresh medium 6 h later, and the cell supernatant was collected and filtered 48 h posttransfection. Vesicular stomatitis virus pseudoparticles (VSVpp) were also generated accordingly to serve as controls.

The entry assay was performed as described previously (Bartosch et al., 2003). Huh7 cells were incubated with HCVpp or VSVpp at 37°C for 6 h before changing the medium. The entry efficiency was evaluated 72 h postincubation by flow cytometry.

### Quantitative Reverse Transcription PCR

RNA was extracted using RNAiso reagent (Takara, Japan) according to the manufacturer’s protocols. Total RNA was quantified at 260 nm with a spectrophotometer and reverse transcribed with a PrimeScript^TM^ RT Master Mix kit (Takara, Japan) according to manufacturer’s instructions. Quantitative reverse transcription PCR (RT-qPCR) was performed using TB Green Premix^®^ Ex Taq^TM^ (Takara, Japan) followed with a Step One real time PCR system (Applied Biosystems, Foster City, CA, United States). GAPDH was used as an mRNA endogenous control to normalize the expression of other mRNAs. The sequences of primers used in the present study are listed in Table 2.

**TABLE 2:** Sequences of primers used in RT-PCR analysis.

Gene	Primer sequence, 5′–3′	
	Forward	Reverse
SNORD126	TGCATGTTAAGTCCGTGTTTCAG	CAGAGCATGTGTTTAATCAGGC
U6	CGCTTCGGCAGCACATATAC	ATTTGCGTGTCATCCTTGCG
HCV RNA	CTGCCCATCCACTGAGACATA	AGCTTGGGGTCATGGCAAAC
GAPDH	CCGGCTTCCACACATCCTTAT	AAGGCCAGTATGCACAGCTT
CLDN	TTGGGCTTCATTCTCGCCTT	GTCGCCGGCATAGGAGTAAA

### IF Microscopy and Quantification

During indirect IF assay, Huh7 cells were fixed with cold methanol at −20°C or 4% paraformaldehyde at room temperature for 30 min before being blocked with 3% bovine serum albumin. HCV core or CLDN1 expression was evaluated with their corresponding primary antibodies and appropriate secondary antibodies. The infection rate or protein expression level was detected using a fluorescence microscope cell imaging system or a confocal laser scanning microscope. Quantification of HCV infection rate in Huh7 cells is conducted by calculating the mean ratio of HCV-positive cells under microscopy of randomly selected six fields in each well using ImageJ software^3^. Quantification of CLDN1 expression was analyzed by calculating the relative fluorescence intensity of each cell in randomly selected six fields in each well using ImageJ software.

### Western Blotting and Flow Cytometry

Cells were lysed with RIPA lysis buffer (Santa Cruz Heidelberg, Germany) on ice, and the total protein concentration was measured with a BCA protein assay kit (Pierce Biotechnology, Rockford, IL, United States) with protease inhibitor cocktail and PMSF (Beyotime, China) added to reduce protein degradation. The expression of HCV entry factors or phosphorylated proteins was analyzed 24 h after cell treatment by Western blot analysis using their corresponding antibodies as described above at recommended concentrations. Quantification of protein expression was evaluated by detecting protein gray scale of three repeated experiments using ImageJ and normalized using the GAPDH level, and calculated as relative value according to the negative control (NC).

Flow cytometry was used to detect HCV entry as mentioned above, and the expression level of CD81 24 h after cell treatment was assessed using its monoclonal antibody (1D6-CD81, APC) according to the manufacturer’s instructions. Briefly, cells were digested with non-EDTA trypsinase, washed with phosphate-buffered saline, resuspended in binding buffer containing the antibody and then incubated in dark for 30 min in centrifuge tubes. The cells were then washed twice before they were analyzed by flow cytometry (Beckman, United States).

### Statistical Analysis

The bar and line graphs are presented with the mean values and standard deviation of at least three independent experiments. Statistical analyses were performed using two-tailed Student’s *t*-test, or an analysis of variance (ANOVA) followed by the Tukey *t*-test was performed for comparison of two groups or multiple groups where appropriate. All statistical analyses were performed using the Statistical Product and Service Solutions (SPSS) version 17.0. *p* < 0.05 was considered to be significant.

## Results

### SnoRNA Profiling Reveals Dysregulated SnoRNAs in Infected Hepatoma Cells

**FIGURE 1:**
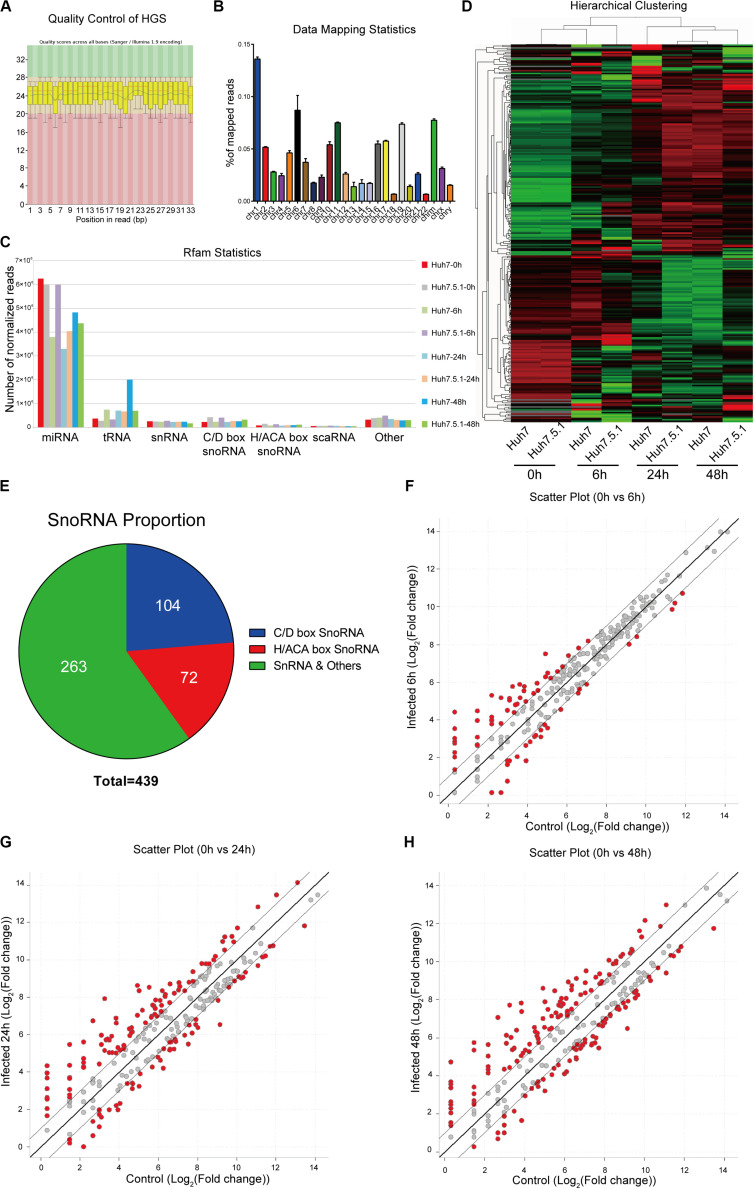
RNA expression profile of high-throughput sequencing during HCV infection. **(A)** Representative images of quality scores of samples during the sequencing. **(B)** Percentage of average mapped reads to each chromosome in all samples. **(C)** The distribution of normalized reads of different small RNAs in each sample. **(D)** Hierarchical clustering of the samples showing the expression pattern. Note that samples of the same time point were clustered together, and the time point was chosen according to the possible life cycle of HCV infection in hepatoma cells. The red color represented relatively upregulated snoRNAs, and green color represented the downregulated snoRNAs. **(E)** The distribution of small RNAs identified in the sequencing data. The scatter plot showing the differentially expressed snoRNAs in entry **(F)**, replication **(G)**, and transmission **(H)** stages of HCV infection.

To assess the small RNA expression patterns during HCV infection, we performed snoRNA profiling using high-throughput RNA sequencing of HCV-infected and non-infected Huh7 and Huh7.5.1 cells. The quality control and genome mapping data confirmed the overall quality of the sequencing data of RNAs smaller than 100 base pairs (Figures 1A,B). From the statistical data of processed Rfam reads, a large number of reads were observed to map to microRNAs, whereas few snoRNAs and other small RNAs were identified, with more C/D box snoRNAs observed than H/ACA box snoRNAs and snRNAs (Figure 1C). Although small in number, the snoRNAs showed similar expression pattern during HCV infection between Huh7 and Huh7.5.1 cells, indicating that they may have active roles during HCV infection (Figure 1D). Altogether, 263 snRNAs and scaRNAs were identified, whereas 104 C/D box snoRNAs and 72 H/ACA box snoRNAs were identified as being expressed in HCV-infected and non-infected Huh7 and Huh7.5.1 cells (Figure 1E). The scatter plot comparing different HCV infection time points with non-infected hepatoma cells showed that in the early (or entry) stage of HCV infection, fewer snoRNAs were dysregulated (1.5-fold change) than that observed during later infection stages (Figures 1F–H). Taken together, these results provided initial data on the snoRNA expression patterns in hepatoma cells during HCV infection and showed that although gradual, the snoRNAs were dysregulated during HCV infection.

### C/D Box SNORD126 Expression Is Significantly Reduced in Infected Hepatoma Cells

**FIGURE 2:**
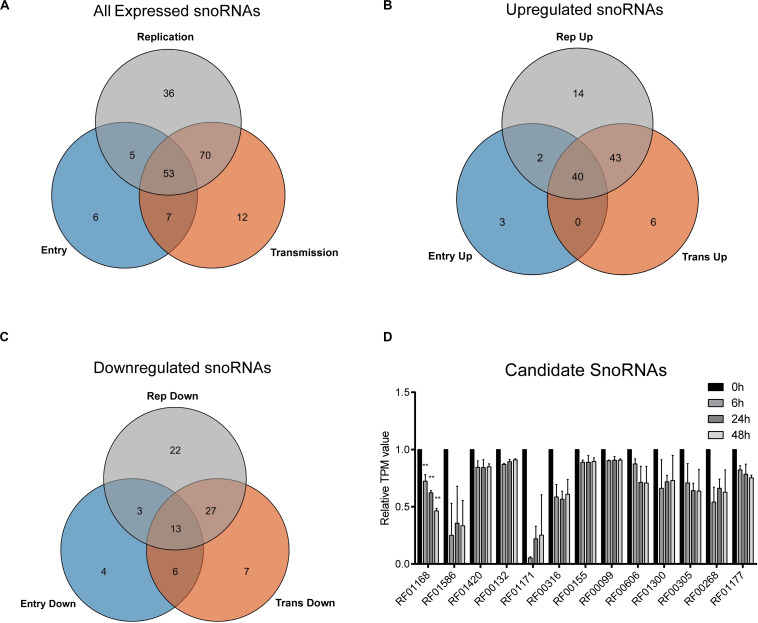
HCV infection-related snoRNA candidates screening. Venn plot showing all **(A)** or upregulated **(B)** or downregulated **(C)** differentially expressed snoRNAs in entry, replication, and transmission stages of HCV infection compared with non-infected cells. **(D)** The TPM (transcript per million reads) values of the 13 all-stage downregulated snoRNAs at different time points according to the sequencing data. Data are shown as mean ± SD. **P < 0.01 compared with the uninfected group.

To gain more insights into the potential role of snoRNAs during HCV infection, we further analyzed the differentially expressed snoRNAs. As we harvested the infected cells according to the time points that could represent different viral life cycle stages for high-throughput RNA sequencing, we roughly divided the differentially expressed snoRNAs into the specific groups entry (0 vs. 6 h), replication (0 vs. 24 h) and transmission (0 vs. 48 h), and compared them using a Venn plot (Figure 2A). The results showed that of the 176 snoRNAs we identified, 53 showed dysregulated expression during all possible viral life cycle stages (Figure 2A). By further defining the upregulated and downregulated snoRNAs, we observed that the expression of 40 was consistently upregulated during all stages compared with that observed in normal hepatoma cells (Figure 2B), whereas only 13 snoRNAs showed consistently downregulated expression during all viral cycles (Figure 2C), indicating the different roles that individual snoRNA may have during HCV infection. Because downregulated snoRNAs may have a negative effect on HCV infection, we chose the 13 downregulated snoRNAs as candidates for further analysis. By evaluating the expression level of these 13 snoRNAs, we observed that RF01168 or SNORD126, a C/D box snoRNA, showed the most drastic expression change during infection and was therefore chosen for further functional analysis (Figure 2D).

### Reduced SNORD126 Expression Is Correlated With Viral Infection Progression and Concentration

**FIGURE 3:**
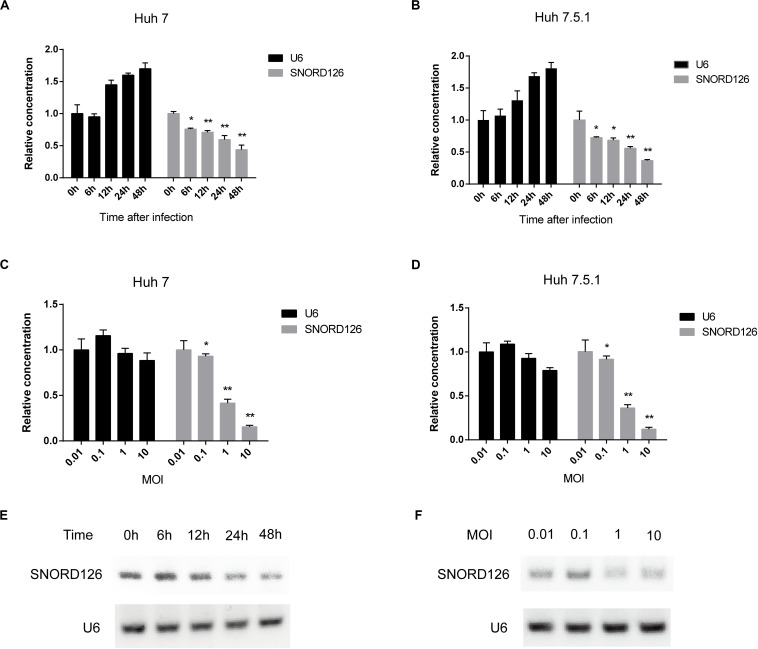
SNORD126 was reduced by HCV infection in Huh7 and Huh7.5.1 cells. **(A)** Huh7 cells and Huh7.5.1 cells **(B)** were infected with HCVcc of JFH-1 (MOI = 1). Then the cells were lysed at different time points after infection, and the expression level of SNORD126 was determined by qPCR. U6 was introduced as an endogenous control. Results were shown as relative concentration. **(C)** Huh7 cells and Huh7.5.1 cells **(D)** were infected with HCVcc of JFH-1 by different MOIs. Then the cells were lysed at 48 h after infection, and the expression level of SNORD126 was detected by qPCR. U6 was introduced as an endogenous control. Results were shown as relative concentration. **(E)** The expression level of SNORD126 was also tested by gel electrophoresis after PCR amplifying by Huh7 cells lysed at different time points after infection, or **(F)** Huh7 cells infected with different MOIs lysed at 48 h after infection. All experiments are repeated at least three times. Data are shown as mean ± SD. *t*-test was performed in all figures which need statistical analysis. *P < 0.05; **P < 0.01 compared with endogenous control.

To further validate that HCV infection affects the SNORD126 expression profile, we infected Huh7 and Huh7.5.1 cells with HCVcc of JFH-1 and examined gene expression at different time points postinfection by RT-qPCR. As shown in Figures 3A,B, SNORD126 expression was significantly reduced along with infection time, while that of the endogenous control U6 gradually increased, as cells continued to proliferate. These results suggested that viral infection progression suppresses SNORD126 expression.

Huh7 and Huh7.5.1 cells were also incubated with HCVcc of different MOIs to evaluate the effect of viral concentration on SNORD126 expression. The RT-qPCR results indicated that SNORD126 was remarkably downregulated as the viral titer increased, whereas U6 expression was slightly reduced, probably due to the cytotoxicity associated with high viral concentrations (Figures 3C,D), further confirming that the viral concentration affected SNORD126 levels.

The SNORD126 expression profile was also verified by agar gel electrophoresis of amplified target genes of different time points postinfection or infection concentrations in Huh7 cells. Consistent with the above RT-qPCR results, the expression of SNORD126 significantly decreased during the latter stage or with higher MOIs (Figures 3E,F). Taken together, these data suggested that SNORD126 expression was reduced due to viral infection progression and concentration.

### SNORD126 Overexpression Promotes HCV Infection

**FIGURE 4:**
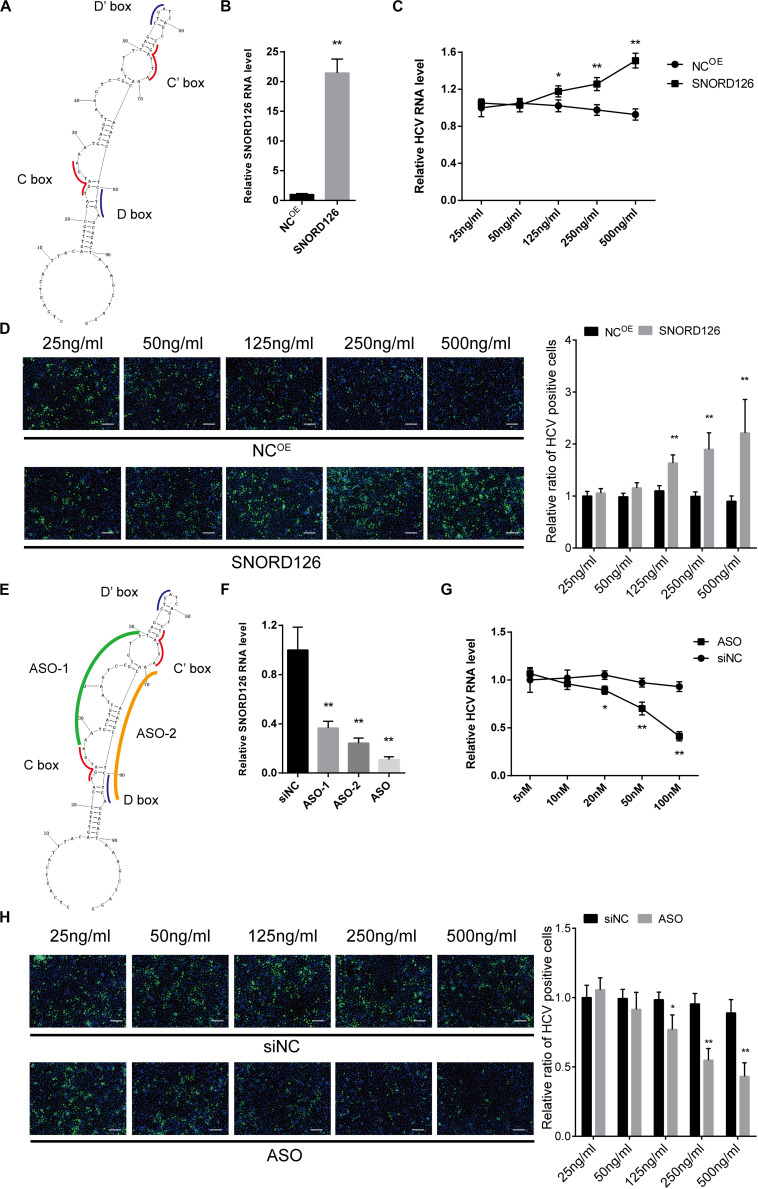
Overexpression and interference of SNORD126 on HCV infection. **(A)** The secondary structures of SNORD126 from 1 to 99 nt. **(B)** The overexpression efficiency of SNORD126 on Huh7 cells was assessed by qPCR 48 h posttransfection of 500 ng/mL full-length plasmid. Results were shown as relative SNORD126 RNA level. Huh7 cells were transfected with indicated concentrations of SNORD126, followed by the infection of JFH-1 HCVcc (MOI = 1). Effects of SNORD126 on HCV infection were evaluated by both qPCR **(C)** and immunofluorescent assay **(D)** 48 h postinfection to test viral RNA level and core protein expression. The bar represents 200 μm. **(E)** Schematic diagram showing the target sites of antisense oligonucleotides. **(F)** Huh7 cells were transfected with 100 nM antisense oligonucleotides of ASO-1, ASO-2, and mixture of ASO-1 and ASO-2 (ASO) targeting SNORD126, and the downregulation efficiency was detected by qPCR. Huh7 cells were transfected with indicated concentrations of ASO, followed by the infection of JFH-1 HCVcc (MOI = 1). HCV infection was evaluated by testing HCV RNA level using qPCR **(G)** and HCV core with immunofluorescent assay **(H)** 48 h postinfection. All experiments were repeated at least three times, and GAPDH is used as an internal control. Data are shown as mean ± SD. *t*-test was performed in all figures that need statistical analysis. *P < 0.05; **P < 0.01 compared with negative control.

To gain insights into SNORD126 expression during HCV infection, we transfected Huh7 cells with a SNORD126 overexpression plasmid (Figure 4A). The overexpression efficiency was tested by RT-qPCR, and the results showed significantly elevated SNORD126 expression after treatment (Figure 4B). Cells were then transfected with the indicated concentration of a SNORD126 overexpression plasmid before they were infected with HCVcc. The gain-of-function assay results revealed that SNORD126 promoted HCV infection in a dose-dependent manner, as indicated by both elevated cellular HCV RNA levels (Figure 4C) and viral core protein expression (Figure 4D) as the concentration of transfected snoRNA increased.

### SNORD126 Knockdown Inhibited HCV Infection

Loss-of-function assays were also performed to investigate the underlying mechanism of SNORD126 activity during HCV infection. We used ASOs that were complementary to the functional motifs of SNORD126 to interfere with its normal functions (Figure 4E). Because these areas are crucial to the binding and catalysis of rRNAs, the utilization of these ASOs would obviously block the snoRNP functions. The RT-qPCR analysis confirmed the significant downregulation effect of ASOs, especially when used as a mixture, on endogenous SNORD126 expression in transfected Huh7 cells (Figure 4F), suggesting that ASOs could impair the functions and the expression of SNORD126.

Subsequently, the effect of SNORD126 inhibition on HCV infection was analyzed. As the concentration of transfected ASOs increased, viral RNA levels significantly decreased compared with that observed in the NC in a dose-dependent manner (Figure 4G). HCV infection was also determined by testing viral core protein levels through an IF assay, and the results showed an obvious decrease in colonies of ASOs-transfected cells compared with that observed in the control groups (Figure 4H).

### SNORD126 Promotes HCV Entry, but Does Not Affect Viral Replication and Release

**FIGURE 5:**
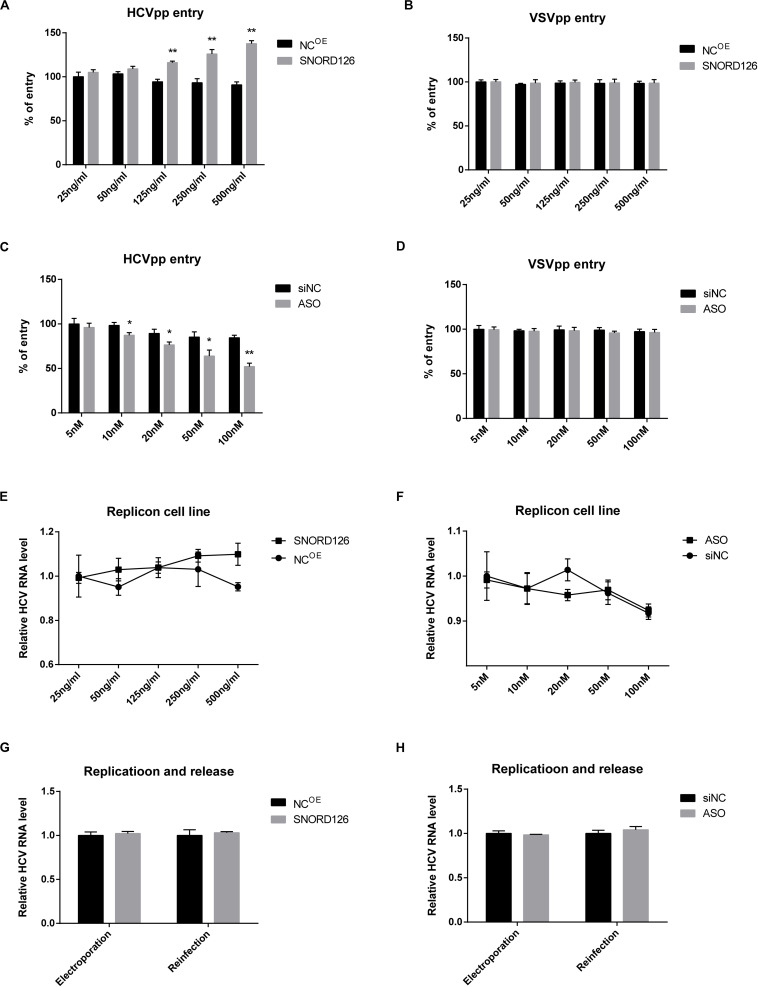
Effect of SNORD126 on different stages of HCV life cycle. Huh7 cells were transfected with indicated concentration of SNORD126 **(A,B)** or ASO **(C,D)** and subsequently inoculated with HCVpp of H77 or VSVpp. The entry of HCVpp or VSVpp was tested by flow cytometry. Results were shown as percentage of entry compared with the negative control, *t*-test. The BB7 replicon cells were transfected with indicated concentrations of SNORD126 **(E)** and ASO **(F)**, and HCV RNA levels were tested by qPCR. SNORD126 overexpression plasmid of 500 ng/mL **(G)** or ASO of 100 nM **(H)** was transfected into Huh7 cells before JHF-1 HCV RNA electroporation. At 48 h postinfection, cells were lysed to detect viral replication efficiency by intracellular HCV RNA level (ANOVA), and supernatants were collected to reinfect naive host cells to evaluate the effect of SNORD126 on release of viral life cycle (*t*-test). All experiments were repeated at least three times, and GAPDH is used as an internal control. Data are shown as mean ± SD. *P < 0.05; **P < 0.01 compared with negative control.

To further determine the period in which SNORD126 promotes HCV infection, we evaluated the role of this snoRNA at different steps of viral life cycle. HCVpps were utilized to test the effect of SNORD126 on viral entry. A SNORD126 overexpression plasmid or its ASOs at indicated concentrations were transfected into Huh7 cells, which were then incubated with HCVpp or vesicular stomatitis VSVpp to detect their ability to enter host cells. As shown in Figures 5A,B, upregulation of SNORD126 could promote HCVpp entry in a dose-dependent manner compared with that observed in the NC group, while the ability of SNORD126 to promote VSVpp infection was not obvious. The interference of SNORD126 by ASOs had the opposite repressive effect on HCVpp entry in a dose-dependent manner, and yet this inhibitory effect was also not detected for VSVpp when compared with that observed in the NC group (Figures 5C,D).

Subsequently, the role of SNORD126 on viral replication and release was examined. HCV replicon cells were used to assess the influence of SNORD126 on viral replication. As shown in Figures 5E,F, neither overexpression nor knockdown of this snoRNA altered HCV RNA levels in replicon cells. Huh7 cells were also electroporated with subgenomic HCV RNA, and intracellular HCV RNA levels and extracellular viral infectivity were assessed to determine whether SNORD126 targets viral replication or release. However, no significant changes were observed even under obvious snoRNA alteration by transfection of either the overexpression plasmid or interfering oligonucleotides, indicating that the inhibitory effect of SNORD126 on HCV infection is not due to viral replication or release stage (Figures 5G,H).

### SNORD126 Boosts HCV Entry by Upregulating the Expression of CLDN1

**FIGURE 6:**
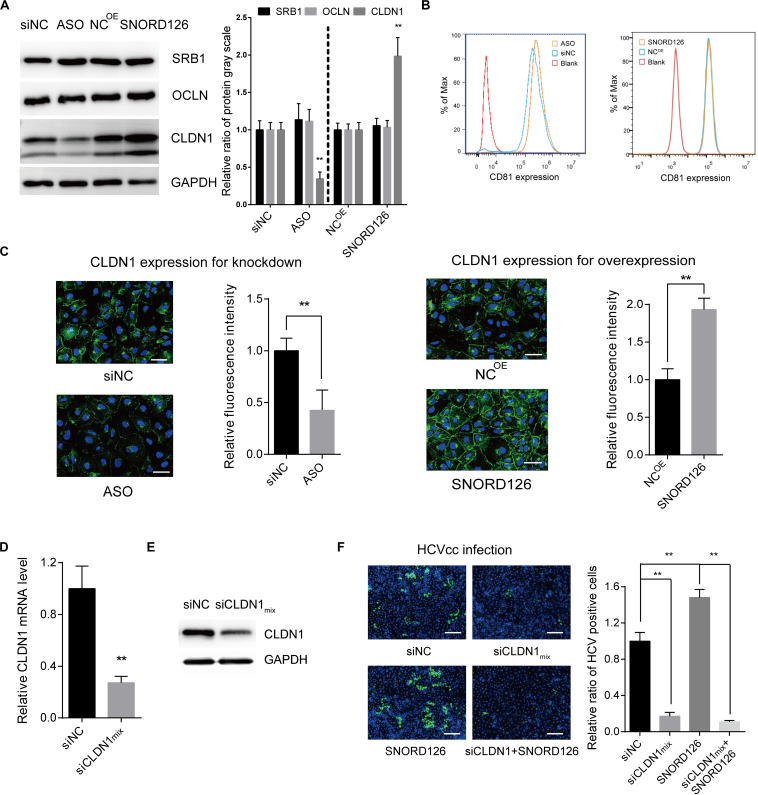
SNORD126 affected the expression of viral entry factor CLDN1. **(A)** Huh7 cells were transfected with 500 ng/mL SNORD126 plasmid or 100 nM ASO, and the expression levels of SRB1, OCLN, CLDN1, and GAPDH were tested by Western blotting, and CD81 by flow cytometry **(B)** 48 h postinfection. The relative ratio of protein gray scale was analyzed. **(C)** CLDN1 expression and localization were determined by confocal microscopy. The bar represents 50 μm. Fluorescence intensity was detected in each well with six different fields randomly and normalized to the relative value. **(D,E)** Verifying CLDN1 knockdown efficiency. Huh7 cells were transfected with mixture of siCLDNs (siCLDN1mix), and CLDN1 expression was detected by qPCR **(D)** and Western blotting **(E)**. **(F)** Huh7 cells were transfection with either or both siCLDN1mix and SNORD126 before being infected by JFH-1 HCVcc. Infection was determined by testing viral core protein level with immunofluorescent assay. The bar represents 200 μm. Ratio was calculated under microscopy by counting positive cells in six different fields randomly in one well. All experiments were repeated at least three times, and GAPDH is used as an internal control. Data are shown as mean ± SD. *t*-test was performed in all figures that need statistical analysis. **P < 0.01 compared with negative control.

To elucidate the mechanism by which SNORD126 promotes HCV entry, we analyzed the effect of SNORD126 on the expression of essential virus entry factors including scavenger receptor class B type I (SRB1), tetraspanin CD81, CLDN1, and occludin (OCLN). Huh7 cells were transfected with either the SNORD126 overexpression plasmid or knockdown oligonucleotides and then assessed for the expression levels of these four entry factors by Western blot and flow cytometry analyses. As shown in Figures 6A,B, SNORD126 did not alter the protein expression of the assayed entry factors except tight junction protein CLDN1. The correlation between SNORD126 and CLDN1 was also evaluated by immunofluorescence analysis, which showed that SNORD126 knockdown reduced CLDN1 expression and disrupted its distribution on the cell surface, whereas SNORD126 overexpression had the opposite effect (Figure 6C).

We further determined the role of CLDN1 in the SNORD126-mediated enhancement of HCV infection through loss-of-function analysis. siRNAs targeting CLDN1 were synthesized, and the efficiency of the siRNA mixture was tested by RT-qPCR and Western blot analyses (Figures 6D,E). As revealed above, SNORD126 promoted virus infection. However, CLDN1 inhibition of viral entry by siRNAs could abrogate this effect, indicating that SNORD126 promotes HCV infection during an early step of the viral life cycle, probably by maintaining the relatively high expression level of essential entry factor CLDN1 (Figure 6F).

### SNORD126 Activates AKT Phosphorylation in SnoRNP Form to Increase CLDN1 Expression

**FIGURE 7:**
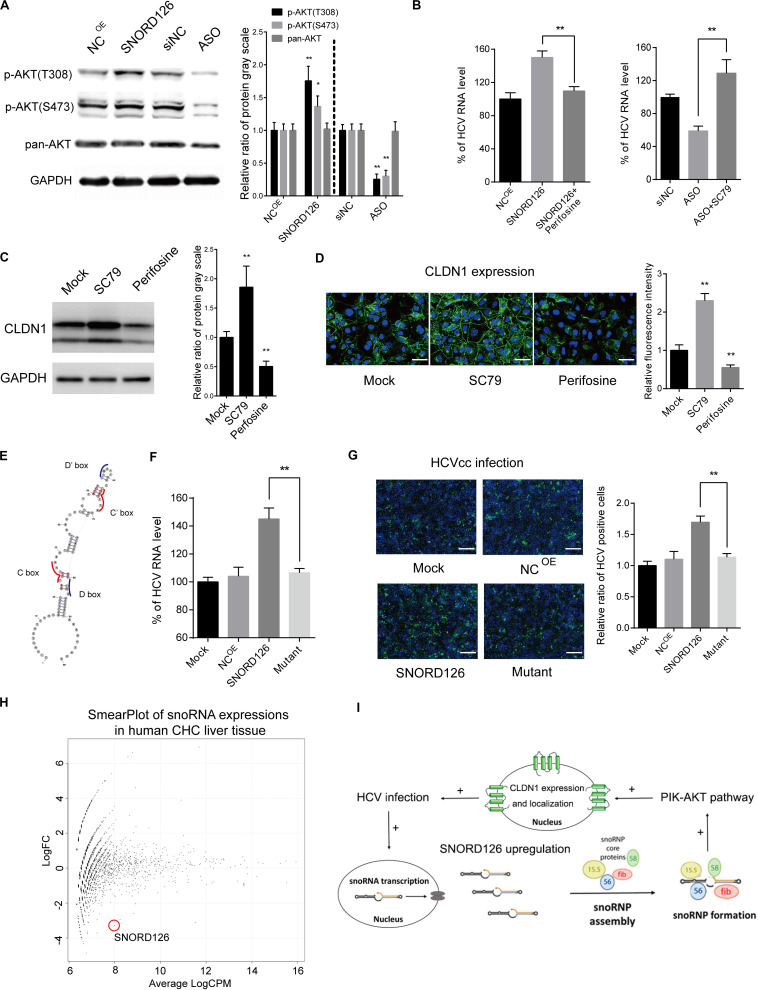
Promotion of CLDN1 expression was mediated by PI3K-AKT pathway through snoRNP function. **(A)** Huh7 cells were transfected with 500 ng/mL SNORD126 plasmid or 100 nM ASO before the expression levels of p-AKT(T308), p-AKT(S473), AKT, and GAPDH were determined by Western blotting. The relative ratio of protein gray scale was analyzed. **(B)** Huh7 cells were treated with perifosine (10 μM) or SC79 (5 μM) before they were transfected with SNORD126 overexpression plasmid (500 ng/mL) or inhibitory ASO (100 nM). Cells were then infected by JFH-1 HCVcc and lysed to detect viral RNA level by qPCR 48 h postinfection. Results were shown as percentage of HCV RNA level. **(C,D)** Huh7 cells were treated with SC79 (5 μM) and perifosine (10 μM), and CLDN1 expression level and localization were assessed by both Western blotting **(C)** and confocal microscopy **(D)**. The bar represents 50 μm. The relative ratio of protein gray scale was analyzed. Fluorescence intensity was detected in each well and normalized to the relative value. **(E)** Schematic diagram showing the mutated sites of SNORD126 to generate C/D box mutated SNORD126 for functional analysis. **(F,G)** Huh7 cells were transfected with SNORD126 or its mutant plasmids before being infected by HCVcc of JFH-1. HCV infection was determined by viral RNA level with qPCR (F) and core protein level with immunofluorescent assay **(G)** 48 h after infection. The bar represents 200 μm. Ratio was calculated under microscopy by counting positive cells in six different fields randomly in one well. The above experiments were repeated three times, and GAPDH was used as an internal control. *t*-test was performed in the above figures that need statistical analysis. Data are shown as mean ± SD. *P < 0.05; **P < 0.01. **(H)** High-throughput sequencing data of GSE87843 were reanalyzed and mapped to human snoRNA sequences in Rfam database, and the smear plot depicting the differentially expressed snoRNAs was shown. Note the red circle labeled snoRNA was SNORD126 in the data. **(I)** A summary of role that SNORD126 may take in promoting HCV infection.

SNORD126 was previously shown to activate the PI3K-AKT pathway to promote carcinoma cell growth. This pathway was also observed to be closely involved in HCV entry and replication processes. Therefore, we wondered whether the ability of SNORD126 to promote viral infection was associated with the activation of this pathway. SNORD126-overexpressing or knockdown Huh7 cells were evaluated by Western blot analysis to elucidate the effect of this snoRNA on the AKT phosphorylation status. As shown in Figure 7A, SNORD126 overexpression significantly enhanced AKT phosphorylation, while interfering oligonucleotides potently suppressed this process. In addition, the AKT inhibitor perifosine could reverse the promotion of HCV infection in SNORD126-overexpressing Huh7 cells (Figure 7B). In contrast, treatment with the AKT activator SC79 increased HCV RNA levels when combined with SNORD126 knockdown oligonucleotides (Figure 7B). To further verify whether the alteration of AKT phosphorylation by SNORD126 influences CLDN1 expression, we treated Huh7 cells with either perifosine or SC79 and assessed CLDN1 protein levels by both Western blot and immunofluorescence analyses. We discovered that SC79 increased the expression and distribution of CLDN1, while perifosine had the opposite effect (Figures 7C,D). Thus, our results showed that SNORD126 affects HCV infection by altering CLDN1 expression levels by affecting AKT phosphorylation.

Subsequently, we attempted to determine whether the ability of SNORD126 to regulate HCV entry was related to the snoRNP mechanism. To this end, we designed a mutant transcript with an altered core protein binding area according to the known structural characteristics (Figure 7E). As shown in Figures 7F,G, the mutant transcript significantly abolished the promoting effect of the wild-type transcript on viral infection with respect to both viral RNA levels and protein expression, further demonstrating that the functions of SNORD126 on HCV infection are snoRNP dependent.

### SNORD126 Is Downregulated in Chronic Hepatitis C Liver Samples

Considering the significant HCV infection promotion effect of SNORD126 *in vitro*, we searched for human liver sequencing data of chronic hepatitis C (CHC) patients. Using the existing GEO (Gene Expression Omnibus) database, we reanalyzed the GSE87843 data and generated the global snoRNA profiles of human CHC liver samples. We found that SNORD126 downregulated significantly in CHC liver samples compared to normal human liver samples (Figure 7H and Supplementary Figure S1), and is consistent with our previous findings.

Together, we discovered altered expression of an oncogenic SNORD126 during HCV infection by high-throughput sequencing analysis and made further study to reveal its probable mechanism of promoting viral entry by increasing CLDN1 expression through activation of the PI3K-AKT pathway. This process is partly associated with SNORD126-mediated snoRNP function (Figure 7I).

## Discussion

HCV infection is a complex pathological process requiring the participation of various viral and host factors. Of all the host factors involved in this process, ncRNAs, which were once thought to be unimportant, have increasingly been shown to have important regulatory functions in a variety of pathological events, including viral infection (Esteller, 2011; Xu et al., 2019). SnoRNAs are ncRNAs for which a number of have been performed to elucidate the molecular mechanisms associated with their activity function (Mourksi et al., 2020; Shuwen et al., 2020). SnoRNAs typically function as snoRNPs, where specific binding sites on RNAs are provided for core proteins to integrate and work as a stable and functional complex (Massenet et al., 2017). The abundances of a number of snoRNAs have been observed to change during several viral infection processes, such as for chikungunya virus, avian influenza virus, and Epstein-Barr virus infection (Hutzinger et al., 2009; Samir et al., 2020; Saxena et al., 2013). SnoRNAs can regulate antiviral responses by acting on relevant essential elements and can also be utilized by viruses to escape natural immunity through modulation of RNA activity. In our present study, we observed the snoRNA SNORD126 to be increasingly downregulated in HCV-infected cells along with viral infection progression by high-throughput RNA sequencing analysis. The goal of the present study was to elucidate the potential role of SNORD126 during this pathological process. This snoRNA was previously found to be elevated in HCC and CRC patient samples and to promote cell growth in these two cell lines *in vitro* (Fang et al., 2017). However, the function and underlying mechanism of SNORD126 activity during HCV infection have not been studied.

According to our data, we obtained compelling evidence that SNORD126 promotes HCV infection during an early stage of the viral entry step by upregulating the expression of the essential HCV entry factor CLDN1. The effect of SNORD126 on CLDN1 was shown to be associated with the phosphorylation of the kinase AKT, a key component of PI3K-AKT signaling pathway that has been reported to play important roles in HCV entry, replication, and translation (Liu et al., 2012; Cheng et al., 2015; Shi et al., 2016). These findings were consistent with those of a previous study in which SNORD126 exhibited oncogenic ability by activating AKT through fibroblast growth factor receptor 2 (Fang et al., 2017). The results showed that SNORD126 tended to function in its snoRNP form instead through a posttranscriptional modification or microRNA-like function, which was demonstrated through several approaches, including the use of SNORD126 knockdown ASOs and a mutant transcript that blocked the binding sites of snoRNP core proteins. However, our current results could not reveal the exact working sequence elements and characteristic secondary structures associated with this process, and the affected molecular network of the SNORD126-based snoRNP was obscure due to a lack of proper specialized approaches. In this regard, novel and advanced methods should be applied in the future to accurately detect how snoRNP affects downstream pathway to elucidate whether it functions through its regular processing and modifying ribosomal RNAs or by another unidentified mechanism.

In addition, a previous study showed that SNORD126 was highly expressed in HCC patient samples. As is known to all, HCV infection is the second leading cause of HCC. Approximately 20% of late-stage HCV patients progress into liver cirrhosis and HCC (Maucort-Boulch et al., 2018; Plissonnier et al., 2018). The contrasting regulatory patterns of SNORD126 between HCC and HCV infection may indicate the existence of an underlying mechanism involved in the etiology or pathological development of HCV infection and liver cancer. At the beginning of viral infection, the host might resist external infection by downregulating factors that facilitate this process. However, once HCV infection progresses into HCC, the cancer cells occupy the leading power and increase the expression of this promoting snoRNA through a specific mechanism to further promote cancer transmission. This intriguing and noteworthy phenomenon requires further investigation in the future. Moreover, the reanalyzed dataset of CHC-infected liver tissue sequencing data further added to the credibility that SNORD126 may be functional in human liver cells. Based on the above inference, SNORD126, which is both an oncogene and a viral infection-promoting element, could serve as a potential diagnostic and therapeutic target during different phases of this pathological process, enhancing the clinical application value of this snoRNA.

HCV entry is the very first step of viral infection, and targeting this initial step could contribute to viral inhibition, as several previous studies have indicated (Tarr et al., 2013; Lin et al., 2015; Elsebai et al., 2016). Moreover, entry inhibitors also have additional effects beyond current DAAs, including a prophylactic vaccine-like effect, the prevention of HCV spread by blocking viral reinfection, and restriction of the breakthrough of DAA-resistant viruses. These unique advantages will benefit end-stage HCV-induced liver disease patients, especially those who need liver transplantations. The combination of entry inhibitors and current DAAs would further promote therapeutic strategies for HCV infection.

In summary, for the first time, we demonstrated that the snoRNA SNORD126 facilitates HCV entry into host cells by influencing the functioning of snoRNP, which promotes the phosphorylation of AKT to increase the CLDN1 expression level. The results of the present study provide novel insights into the function and underlying molecular mechanism of SNORD126 in HCV infection, expanding the understanding of how host factors such as snoRNAs promote viral invasion and how the human body interferes with this process. Therefore, our findings indicate that snoRNAs are potential prospective diagnostic biomarkers or therapeutic targets for pathological processes such as HCV infection.

## Data Availability Statement

The datasets generated for this study can be found in the GEO, with accession number GSE156205. The data are accessed using the following link https://www.ncbi.nlm.nih.gov/geo/query/acc.cgi?acc=GSE156205.

## Author Contributions

XQ, PZ, and ZQ designed the research. XQ and CX analyzed the data. XQ, CX, and BW performed the research. XQ wrote the manuscript. CX contributed to the proofreading of the manuscript. All authors contributed to the article and approved the submitted version.

## Conflict of Interest

The authors declare that the research was conducted in the absence of any commercial or financial relationships that could be construed as a potential conflict of interest.

## Footnotes

1 https://rfam.org/

2 https://www.ncbi.nlm.nih.gov/geo/

3 https://imagej.nih.gov/

## Supplementary Material

FIGURE S1 The relative TPM expression level of SNORD126 in human liver samples of normal and chronic hepatitis C infected patients were compared. ^∗^*P* < 0.05, Mann–Whitney test.

## Abbreviations

ASO: Antisense oligonucleotide
CLDN1: Claudin-1
DAA: Direct acting antiviral
HCC: Hepatocellular carcinoma
HCV: Hepatitis C virus
NcRNA: Non-coding RNA
OCLN: Occludin
RNP: Ribonucleoprotein
SNORD126, Small nucleolar RNA: C/D box 126
snoRNA: Small nucleolar RNA
SRB1: Scavenger receptor class B type I.

## References

[B1] Bartosch B. Dubuisson J. Cosset F. L. (2003). Infectious hepatitis C virus pseudo-particles containing functional E1-E2 envelope protein complexes. *J. Exp. Med.* 197 633–642. 10.1084/jem.20021756 12615904 PMC2193821

[B2] Boivin V. Deschamps-Francoeur G. Scott M. S. (2018). Protein coding genes as hosts for noncoding RNA expression. *Semin. Cell Dev. Biol.* 75 3–12. 10.1016/j.semcdb.2017.08.016 28811264

[B3] Cavaille J. (2017). Box C/D small nucleolar RNA genes and the Prader-Willi syndrome: a complex interplay. *Wiley Interdiscipl. Rev. RNA* 8:wrna.1417. 10.1002/wrna.1417 28296064

[B4] Cheng D. Zhang L. Yang G. Zhao L. Peng F. Tian Y. (2015). Hepatitis C virus NS5A drives a PTEN-PI3K/Akt feedback loop to support cell survival. *Liver* 35 1682–1691. 10.1111/liv.12733 25388655

[B5] Elsebai M. F. Koutsoudakis G. Saludes V. Perez-Vilaro G. Turpeinen A. M. Fontaine-Vive F. (2016). Pan-genotypic Hepatitis C virus inhibition by natural products derived from the wild egyptian artichoke. *J. Virol.* 90 1918–1930. 10.1128/jvi.02030-15 26656684 PMC4734011

[B6] Esteller M. (2011). Non-coding RNAs in human disease. *Nat. Rev. Genet.* 12 861–874. 22094949 10.1038/nrg3074

[B7] Fang X. Yang D. Luo H. Wu S. Dong W. Xiao J. (2017). SNORD126 promotes HCC and CRC cell growth by activating the PI3K-AKT pathway through FGFR2. *J. Mol. Cell Biol.* 9 243–255. 27913571 10.1093/jmcb/mjw048

[B8] Ferreira A. R. Ramos B. Nunes A. Ribeiro D. (2020). Hepatitis C virus: evading the intracellular innate immunity. *J. Clin. Med.* 9:790. 10.3390/jcm9030790 32183176 PMC7141330

[B9] Hutzinger R. Feederle R. Mrazek J. Schiefermeier N. Balwierz P. J. Zavolan M. (2009). Expression and processing of a small nucleolar RNA from the Epstein-Barr virus genome. *PLoS Pathog.* 5:e1000547. 10.1371/journal.ppat.1000547 19680535 PMC2718842

[B10] Kanwal F. Kramer J. Asch S. M. Chayanupatkul M. Cao Y. El-Serag H. B. (2017). Risk of hepatocellular cancer in HCV patients treated with direct-acting antiviral agents. *Gastroenterology* 153 996–1005.e1001. 28642197 10.1053/j.gastro.2017.06.012

[B11] Li D. K. Chung R. T. (2019). Overview of direct-acting antiviral drugs and drug resistance of Hepatitis C Virus. *Methods Mol. Biol.* 1911 3–32. 10.1007/978-1-4939-8976-8_1 30593615

[B12] Li G. He Y. Liu X. Zheng Z. Zhang M. Qin F. (2017). Small nucleolar RNA 47 promotes tumorigenesis by regulating EMT markers in hepatocellular carcinoma. *Minerva Med.* 108 396–404. 28466632 10.23736/S0026-4806.17.05132-1

[B13] Lin L. T. Chung C. Y. Hsu W. C. Chang S. P. Hung T. C. Shields J. (2015). Saikosaponin b2 is a naturally occurring terpenoid that efficiently inhibits hepatitis C virus entry. *J. Hepatol.* 62 541–548. 10.1016/j.jhep.2014.10.040 25450204

[B14] Liu Z. Tian Y. Machida K. Lai M. M. Luo G. Foung S. K. (2012). Transient activation of the PI3K-AKT pathway by hepatitis C virus to enhance viral entry. *J. Biol. Chem.* 287 41922–41930. 10.1074/jbc.m112.414789 23095753 PMC3516739

[B15] Manns M. P. Buti M. Gane E. Pawlotsky J. M. Razavi H. Terrault N. (2017). Hepatitis C virus infection. *Nat. Rev. Dis. Prim.* 3:17006. 10.1038/nrdp.2017.6 28252637

[B16] Massenet S. Bertrand E. Verheggen C. (2017). Assembly and trafficking of box C/D and H/ACA snoRNPs. *RNA Biol.* 14 680–692. 10.1080/15476286.2016.1243646 27715451 PMC5519232

[B17] Matera A. G. Terns R. M. Terns M. P. (2007). Non-coding RNAs: lessons from the small nuclear and small nucleolar RNAs. *Nat. Rev. Mol. Biol.* 8 209–220. 10.1038/nrm2124 17318225

[B18] Maucort-Boulch D. de Martel C. Franceschi S. Plummer M. (2018). Fraction and incidence of liver cancer attributable to hepatitis B and C viruses worldwide. *Int. J. Cancer* 142 2471–2477. 10.1002/ijc.31280 29388206

[B19] Mourksi N. E. Morin C. Fenouil T. Diaz J. J. Marcel V. (2020). snoRNAs offer novel insight and promising perspectives for lung cancer understanding and management. *Cells* 9:541. 10.3390/cells9030541 32111002 PMC7140444

[B20] Pawlotsky J. M. (2016). Hepatitis C virus resistance to direct-acting antiviral drugs in interferon-free regimens. *Gastroenterology* 151 70–86. 10.1053/j.gastro.2016.04.003 27080301

[B21] Peng X. Li Y. Walters K. A. Rosenzweig E. R. Lederer S. L. Aicher L. D. (2009). Computational identification of hepatitis C virus associated microRNA-mRNA regulatory modules in human livers. *BMC Genom.* 10:373. 10.1186/1471-2164-10-373 19671175 PMC2907698

[B22] Plissonnier M. L. Herzog K. Levrero M. Zeisel M. B. (2018). Non-coding RNAs and Hepatitis C Virus-induced hepatocellular carcinoma. *Viruses* 10:591. 10.3390/v10110591 30380697 PMC6265700

[B23] Qian X. Xu C. Zhao P. Qi Z. (2016). Long non-coding RNA GAS5 inhibited hepatitis C virus replication by binding viral NS3 protein. *Virology* 492 155–165. 10.1016/j.virol.2016.02.020 26945984

[B24] Qian X. J. Zhang X. L. Zhao P. Jin Y. S. Chen H. S. Xu Q. Q. (2016). A schisandra-derived compound schizandronic acid inhibits entry of Pan-HCV genotypes into human hepatocytes. *Sci. Rep.* 6:27268. 10.1038/srep27268 PMC4890123 27252043

[B25] Roingeard P. Beaumont E. (2020). Hepatitis C vaccine: 10 good reasons for continuing. *Hepatology* 71 1845–1850. 10.1002/hep.31182 32060946

[B26] Samir M. Vidal R. O. Abdallah F. Capece V. Seehusen F. Geffers R. (2020). Organ-specific small non-coding RNA responses in domestic (Sudani) ducks experimentally infected with highly pathogenic avian influenza virus (H5N1). *RNA Biol.* 17 112–124. 10.1080/15476286.2019.1669879 31538530 PMC6948974

[B27] Saxena T. Tandon B. Sharma S. Chameettachal S. Ray P. Ray A. R. (2013). Combined miRNA and mRNA signature identifies key molecular players and pathways involved in chikungunya virus infection in human cells. *PLoS One* 8:e79886 10.1371/journal.ppat.079886 PMC3836776 24278205

[B28] Shi Q. Hoffman B. Liu Q. (2016). PI3K-Akt signaling pathway upregulates hepatitis C virus RNA translation through the activation of SREBPs. *Virology* 490 99–108. 10.1016/j.virol.2016.01.012 26855332

[B29] Shuwen H. Xi Y. Quan Q. Yin J. Miao D. (2020). Can small nucleolar RNA be a novel molecular target for hepatocellular carcinoma? *Gene* 733:144384. 10.1016/j.gene.2020.144384 31978508

[B30] Simmons B. Saleem J. Hill A. Riley R. D. Cooke G. S. (2016). Risk of late relapse or reinfection with Hepatitis C virus after achieving a sustained virological response: a systematic review and meta-analysis. *Clin. Infect. Dis.* 62 683–694. 10.1093/cid/civ948 26787172 PMC4772843

[B31] Spearman C. W. Dusheiko G. M. Hellard M. Sonderup M. (2019). Hepatitis C. *Lancet* 394 1451–1466. 31631857 10.1016/S0140-6736(19)32320-7

[B32] Spengler U. (2018). Direct antiviral agents (DAAs) - A new age in the treatment of hepatitis C virus infection. *Pharmacol. Therap.* 183 118–126. 10.1016/j.pharmthera.2017.10.009 29024739

[B33] Stanaway J. D. Flaxman A. D. Naghavi M. Fitzmaurice C. Vos T. Abubakar I. (2016). The global burden of viral hepatitis from 1990 to 2013: findings from the global burden of disease study 2013. *Lancet* 388 1081–1088. 27394647 10.1016/S0140-6736(16)30579-7 PMC5100695

[B34] Stepanov G. A. Filippova J. A. Komissarov A. B. Kuligina E. V. Richter V. A. Semenov D. V. (2015). Regulatory role of small nucleolar RNAs in human diseases. *Biomed. Res. Intern.* 2015:206849. 10.1155/2015/206849 PMC4427830 26060813

[B35] Tarr A. W. Lafaye P. Meredith L. Damier-Piolle L. Urbanowicz R. A. Meola A. (2013). An alpaca nanobody inhibits hepatitis C virus entry and cell-to-cell transmission. *Hepatology* 58 932–939. 10.1002/hep.26430 23553604

[B36] Wakita T. Pietschmann T. Kato T. Date T. Miyamoto M. Zhao Z. (2005). Production of infectious hepatitis C virus in tissue culture from a cloned viral genome. *Nat. Med.* 11 791–796. 10.1038/nm1268 15951748 PMC2918402

[B37] Waziry R. Hajarizadeh B. Grebely J. Amin J. Law M. Danta M. (2017). Hepatocellular carcinoma risk following direct-acting antiviral HCV therapy: a systematic review, meta-analyses, and meta-regression. *J. Hepatol.* 67 1204–1212. 10.1016/j.jhep.2017.07.025 28802876

[B38] World Health Organization [WHO] (2020). Available online at: http://www.who.int/news-room/fact-sheets/detail/hepatitis-c (accessed May 8, 2020).

[B39] World Health Organization [WHO] (2017). *Global Hepatitis Report 2017.* Geneva: World Health Organization.

[B40] Xiong Y. Yuan J. Zhang C. Zhu Y. Kuang X. Lan L. (2015). The STAT3-regulated long non-coding RNA Lethe promote the HCV replication. *Biomed. Pharmacother.* 72 165–171. 10.1016/j.biopha.2015.04.019 26054691

[B41] Xu C. Chen Y. Zhang H. Chen Y. Shen X. Shi C. (2016). Integrated microRNA-mRNA analyses reveal OPLL specific microRNA regulatory network using high-throughput sequencing. *Sci. Rep.* 6:21580. 10.1038/srep21580 PMC4751494 26868491

[B42] Xu Y. Wu W. Han Q. Wang Y. Li C. Zhang P. (2019). New insights into the interplay between non-coding RNAs and RNA-binding protein HnRNPK in regulating cellular functions. *Cells* 8:62. 10.3390/cells8010062 30658384 PMC6357021

[B43] Yang J. H. Zhang X. C. Huang Z. P. Zhou H. Huang M. B. Zhang S. (2006). snoSeeker: an advanced computational package for screening of guide and orphan snoRNA genes in the human genome. *Nucleic Acids Res.* 34 5112–5123. 10.1093/nar/gkl672 16990247 PMC1636440

[B44] Zhang Y. Xu C. Gu D. Wu M. Yan B. Xu Z. (2017). H/ACA box small nucleolar RNA 7A promotes the self-renewal of human umbilical cord mesenchymal stem cells. *Stem Cells* 35 222–235. 10.1002/stem.2490 27573912

[B45] Zhong J. Gastaminza P. Cheng G. Kapadia S. Kato T. Burton D. R. (2005). Robust hepatitis C virus infection in vitro. *Proc. Natl. Acad. Sci. U.S.A.* 102 9294–9299. 15939869 10.1073/pnas.0503596102 PMC1166622

