# Supplementary figures and images for: SNORD126 Promotes Hepatitis C Virus Infection by Upregulating Claudin-1 via Activation of PI3K-AKT Signaling Pathway
Source: Front Microbiol. 2020 Sep 15;11:565590. doi: 10.3389/fmicb.2020.565590 (PMC7522514; doi:10.3389/fmicb.2020.565590)

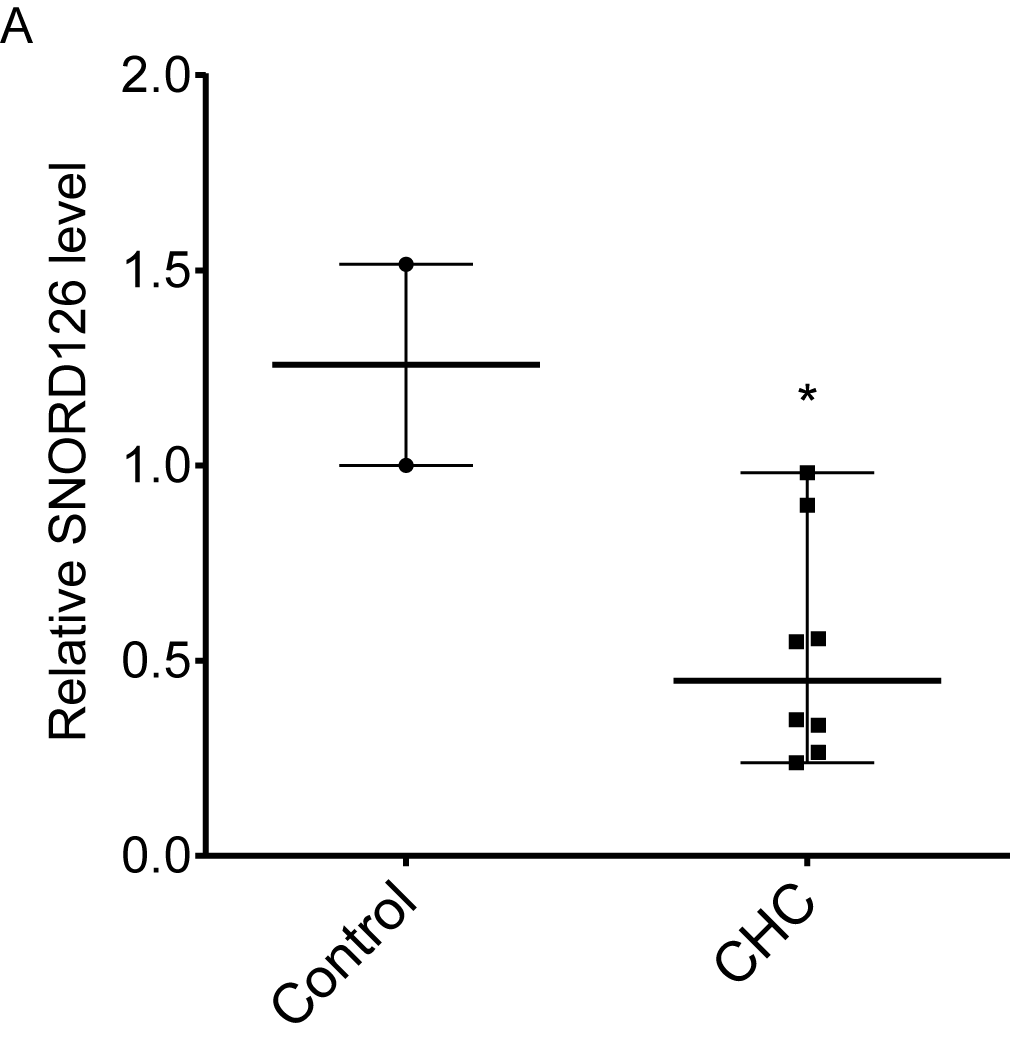

Supplement: FIGURE S1 — The relative TPM expression level of SNORD126 in human liver samples of normal and chronic hepatitis C infected patients were compared. ∗P < 0.05, Mann–Whitney test. [file Image_1.TIF]
